# Supplementary material for: A scalable platform for the development of cell-type-specific viral drivers
Source: eLife. 2019 Sep 23;8:e48089. doi: 10.7554/eLife.48089 (PMC6776442; doi:10.7554/eLife.48089)
Supplement: Supplementary file 4. [file elife-48089-supp4.docx]

| **Supplementary File 4**  Electrophysiological Parameters of GRE44- and GRE44+ SST Neurons in Visual Cortex | | | |
| --- | --- | --- | --- |
|  | **GRE44-** (n=16) | **GRE44+** (n=16) | **p value (2-tailed unpaired t-test)** |
| V_rest_ (mV) | -62.4 ± 1.51 | -60.6 ± 1.63 | 0.41 |
| R_in_ (MΩ) | 304 ± 54.8 | 391 ± 47.3 | 0.24 |
| τ_m_ (ms) | 14.2 ± 2.35 | 22.8 ± 4.32 | 0.094 |
| Threshold (mV) | -45.6 ± 1.24 | -48.1 ± 1.26 | 0.17 |
| AP Peak (mV) | 13 ± 3.38 | 11.6 ± 3.19 | 0.76 |
| AP Trough (mV) | -63.6 ± 1.36 | -63.7 ± 1.43 | 0.96 |
| AP Height (mV) | 76.5 ± 4.46 | 75.2 ± 4.06 | 0.83 |
| **Rate of Rise (V/s)** | **122 ± 11.7** | **85 ± 7.34** | **0.013*** |
| **Rheobase (pA)** | **43.5 ± 10.4** | **20.3 ± 4.64** | **0.044*** |
| **Spike Half-Width (ms)** | **1.25 ± 0.0819** | **2.52 ± 0.307** | **0.0004***** |
| **F_max_, steady-state (Hz)** | **83.4 ± 9.8** | **34.5 ± 4.32** | **0.0002***** |
| **F_max_, initial (Hz)** | **111 ± 8.35** | **67.3 ± 7.48** | **0.0007***** |
| Spike adaptation ratio | 0.763 ± 0.0839 | 0.561 ± 0.0699 | 0.08 |

*Values are shown as mean ± SEM.*
